# The Oxford Tooth Co.

**Published:** 1883-10

**Authors:** 


Under this head we will undertake, either personally or by some one of our associates, to examine and report upon such new inventions appliances and materials as may be submitted to us. Articles will be received and returned at the expense of the owners, but full directions for such return must accompany them. They will be carefully used, but we cannot be responsible for any possible loss or damage.

### THE OXFORD TOOTH CO.

Nearly twenty years ago Dr. C. H. Eccleston, of Oxford, N. Y., left with us a box of teeth manufactured by him, to be either used or returned. Probably the latter would have been their fate, for they did not present an attractive appearance upon the wax, but one day in our inability to find just what we wanted elsewhere, we went to the box and made a closer examination of its contents. This convinced us that some of the moulds were anatomically more correct than any that we had then found, and we commenced their use. During all the vicissitudes of an average lifetime practice they have maintained their place in our laboratory — when we have had one — and some of the moulds we would not willingly do without. We do not use them exclusively, but when we do want them we want them badly. They are very strong, and in metal work especially we have found that they withstand the changes of temperature equal to any that we ever used.

We say not this for Dr. Eccleston's benefit, for we have always paid our bills with him and owe him nothing, but when we have found a man who in all the business dealings of an average business lifetime has not disappointed our just expectations, others have an interest in the information, and therefore we make it public.

